# Transmission of tuberculosis in rural Henan, China: a prospective population-based genomic spatial epidemiological study

**DOI:** 10.1080/22221751.2024.2399273

**Published:** 2024-08-29

**Authors:** Zhuo Quan, Jiying Xu, Meng Li, Changyu Cheng, Peierdun Mijiti, Qi Jiang, Howard Takiff, Zhenhuan Ren, Qian Gao

**Affiliations:** a Shanghai Institute of Infectious Disease and Biosecurity, Key Laboratory of Medical Molecular Virology (MOE/ NHC/CAMS), School of Basic Medical Science, Fudan University, Shanghai, People’s Republic of China; b Institution for Tuberculosis Prevention and Control, Henan Provincial Center for Disease Control and Prevention, Zhengzhou, People’s Republic of China; c Linzhou City Center for Disease Control and Prevention, Anyang, People’s Republic of China; d Department of Epidemiology and Biostatistics, School of Public Health, Wuhan University, Wuhan, People’s Republic of China; e Laboratorio de Genética Molecular, CMBC, Instituto Venezolano de Investigaciones Científicas, IVIC, Caracas, Venezuela

**Keywords:** Tuberculosis, transmission patterns, rural China, whole-genome sequencing, spatial analysis

## Abstract

The incidence of tuberculosis (TB) has declined more slowly in rural than urban areas in China, and data on the patterns of transmission and the high-risk populations in rural areas remains scarce. We conducted a population-based study of culture-positive pulmonary TB patients diagnosed in rural Linzhou City, Henan Province from July 2018 to February 2023. Genomic clusters were defined based on whole-genome sequencing and risk factors for clustering were identified by logistic regression. Transmission events were inferred with phybreak and transmission links were sought through epidemiological investigation of clustered patients. Logistic regression was used to explore the relationship between genomic differences of patient isolates and geographical distances of patient residences. Spatial hotspots were defined using kernel density estimation. Of 455 culture-positive patients, 430 were included in the final analysis. Overall, 192 (44.7%,192/430) patients were grouped into 49 clusters. Clusters containing ≥5 patients accounted for 18.4% (9/49) of the clusters and clustering was highest in student patients. No super-spreaders were detected. Confirmed epidemiologic links were identified for only 18.2% of clustered patients. The clustering risk decreased rapidly with increasing distances between patient residences, but 77.6% of clustered patient pairs lived ≥5.0 km apart. Both the Central Subdistrict and Rencun Township were identified as hotspots for TB transmission. Recent transmission appears to be an important driver of the TB burden in Linzhou. The formulation of effective strategies to reduce TB incidence in rural areas will require further studies to identify high-risk populations and venues where local inhabitants congregate and transmit the infection.

## Introduction

Tuberculosis (TB) remains an important threat to global health, with an estimated 10.6 million people worldwide falling ill and 1.3 million deaths in 2022 [1]. China has the third highest TB burden worldwide and in 2022 had an estimated 750,000 new TB cases and 30,000 TB-related deaths [1]. However, the TB prevalence in the country is not uniform, as the fifth national epidemiological survey reported that the TB burden in rural areas was significantly higher than in urban areas [2]. Furthermore, although the overall incidence of TB in China has shown an impressive decline over the past 20 years, the decline has been less marked in rural compared to urban areas [3]. For example, in Henan, one of the China’s provinces with a high TB burden, the reported incidence showed a decrease from 91.4 per 100,000 to 58.5 per 100,000. In Henan’s rural areas, by contrast, the reduction was only from 108.5 per 100,000 to 90.7 per 100,000, representing just 37% of the decrease reported in Henan’s urban areas [4]. Therefore, a critical challenge for TB prevention and control efforts in China is finding effective strategies to rapidly reduce the TB burden in rural areas.

There are two main pathways for the development of tuberculosis disease – recent transmission and endogenous reactivation. Cases caused by recent transmission generally develop the disease within six months to two years after being infected with *Mycobacterium tuberculosis* (MTB), the bacteria that causes tuberculosis. However, 90–95% of MTB infected individuals will either clear the infection by themselves or become latently infected, and in only 5–10% will the latent infection reactivate to cause disease at some time in the future [5]. Understanding the relative contributions of recent transmission versus endogenous reactivation in high prevalence areas is essential for developing effective prevention and control strategies [5,6]. If the majority of TB cases result from endogenous reactivation, the focus should be on identifying latently infected individuals for prophylactic chemotherapy or careful monitoring. Alternatively, if most of the local TB burden results from recent transmission, control efforts should focus on characterizing local transmission patterns and finding effective strategies to interrupt them [5].

Molecular epidemiology can identify patients with MTB isolates that are very similar, or clustered, and therefore likely to belong to the same chain of recent transmission. Additionally, molecular epidemiology can help identify populations and geographic settings associated with a high-risk of transmission that can be targeted for prevention and control strategies. In San Francisco, for example, strategies targeting populations at high-risk for TB transmission, such as HIV-infected individuals, achieved a notable reduction in TB incidence and clustering from 51.2 per 100,000 and 20.3%, respectively, in 1992 to 19.8 per 100,000 and 12.8% in 1997 [7]. Molecular epidemiological studies conducted in several rural areas in China have suggested that recent transmission is a significant contributor to the rural TB burden. However, very few studies have defined the high-risk populations and the local geographic foci associated with transmission in rural areas of China [8-12]. In this report we combined genomic, epidemiological, and spatial analyses to delineate the patterns of TB transmission in Linzhou city in Henan province.

## Methods

### Study design and population

Linzhou, a county-level city located in the northwest of Henan Province, has an area of 2046 square kilometers. The study included 4 subdistricts and 16 townships in Linzhou (Figure S1A), which in 2020 had an estimated 950,000 inhabitants. The average annual reported TB incidence, from 2018 to 2023, was 66.6 per 100,000 (Figure S1B). The Linzhou City Centre for Disease Control and Prevention (CDC) is responsible for the diagnosis and treatment of TB within the region. Individuals with TB-like symptoms or abnormal chest radiographs in general hospitals and township health centres are referred to the Linzhou CDC, where sputum specimens from patients suspected of having TB are collected for smear microscopy, culture, and rapid molecular tests. Prior to July 2020, sputum specimens were cultured on Löwenstein-Jensen solid media but subsequently culturing was performed using liquid media. The study population included all culture-positive pulmonary TB patients diagnosed between 1 July 2018 and 28 February 2023. The study was approved by the bioethics commission of the Institutes of Biomedical Sciences, Fudan University and all enrolled patients provided informed written consent.

### Whole-genome sequencing

Whole-genome sequencing (WGS) was performed on pre-treatment clinical isolates. For isolates grown in liquid media, the cultures were inoculated onto solid media for amplification. After achieving adequate growth, the isolates were scraped into centrifuge tubes and inactivated at 80°C for 30 min. DNA was extracted using the cetyl-trimethyl ammonium bromide (CTAB) method and sequenced as described [13]. A previously validated pipeline was used to identify single nucleotide polymorphisms (SNPs) [13]. In brief, raw sequence reads were trimmed with Sickle (version 1.33) and aligned to the inferred *Mycobacterium tuberculosis* complex ancestor sequence [14] using BWA-MEM (version 0.7.15). SAMtools (version 1.3.1) and Varscan (version 2.3.6) were then used to identify SNPs. Pairwise SNP distances were calculated based on the fixed SNPs with a frequency ≥75%, excluding those in the repetitive regions of the genome (e.g. PPE/PE-PGRS family genes, phage sequences, and insertions or mobile genetic elements). A genomic cluster was defined as strains differing by 12 or fewer SNPs, consistent with linkage through recent transmission [15].

Based on the identified SNPs, a phylogeny tree was constructed with RAxML-NG (version 1.0.2), using the maximum likelihood method with 100 bootstraps and visualized with Interactive Tree of Life (https://itol.embl.de/). Strain lineage identification and prediction of drug-resistance profiles were obtained using an online platform (https://samtb.uni-medica.com) [16]. Pan-susceptible TB was defined as strains without mutations associated with resistance to any of 17 anti-tuberculosis drugs. Multidrug-resistant (MDR) and pre-extensively drug-resistant (pre-XDR) TB were defined according to the new WHO definitions [1,17]. Other drug-resistant strains were termed other drug-resistant (DR) TB.

### Transmission inference

Transmission directions for each genomic cluster were inferred using phybreak (version 0.5.2) in R [18]. The priors used for mutation rate, the gamma-distributed generation and sampling time were as previously described [19]. We ran 20 independent Markov Chain Monte Carlo (MCMC) simulations with a burn-in of 10,000 cycles and sampling of the independent chains every 50,000 cycles to ensure that most estimated parameters reached an effective sample size >200. Transmission directions with posterior probability >0.5 were included in subsequent analyses.

### Epidemiological investigation

We conducted two epidemiological investigations on the TB patients in the study. First, at the time of TB diagnosis, we routinely collected demographic, clinical, laboratory, and close contact information on each patient. Then, for patients belonging to genomic clusters, we also conducted more in-depth epidemiological investigations. Information was collected on their residence, workplaces and the social settings frequented in the three years prior to TB diagnosis, with particular attention to contacts with other patients within the same cluster. The epidemiological links were defined as confirmed – patients who knew each other and had a history of contact before diagnosis, or probable – patients who did not know each other but shared a setting with high transmission potential (e.g. they lived in the same village or attended the same school).

### Spatial analysis

For all possible pairs of TB patients, we calculated the geographical distance between the residences given by the patients at the time of diagnosis, using the geographic (version 1.5) package in R. We estimated the SNP differences between all pairs of MTB isolates and analysed the distribution of all genomic-clustered patient pairs (patients whose isolates differed by 12 or fewer SNPs) across different geographic distances. We then categorized these clustered patient pairs into intra-township and cross-township groups based on the patient residences. The four subdistricts located in the central area of Linzhou City (Kaiyuan, Guiyuan, Zhenlin, and Longshan subdistricts) were collectively defined as the Central Subdistrict. We evaluated the SNP differences at different geographic distances between the patient residences. Logistic regression was then used to calculate the odds ratios (OR) and 95% confidence intervals (CIs) for genomic clustering at different geographic proximities, with a distance of ≥20 km as the reference [20]. Finally, Kernel density estimation and spatial visualization were performed in ArcGIS (version 10.2) [21].

### Statistical analysis

Non-normal continuous data was expressed as medians and interquartile ranges (IQR), while categorical variables were described using proportions. Differences between groups were tested using the Wilcoxon rank sum test or the chi-square test. Logistic regression was used to calculate the OR and 95% CI for risk factors associated with genomic clustering. Variables with *p*-values less than 0.2 in the univariable analysis were included in the multivariable analysis to calculate the adjusted odds ratios (aOR). Factors with a *p*-value less than 0.05 in the final model were considered statistically significant. All statistical analyses were performed in Stata version 16.0.

## Results

### Characteristics of study population

**Figure 1.**
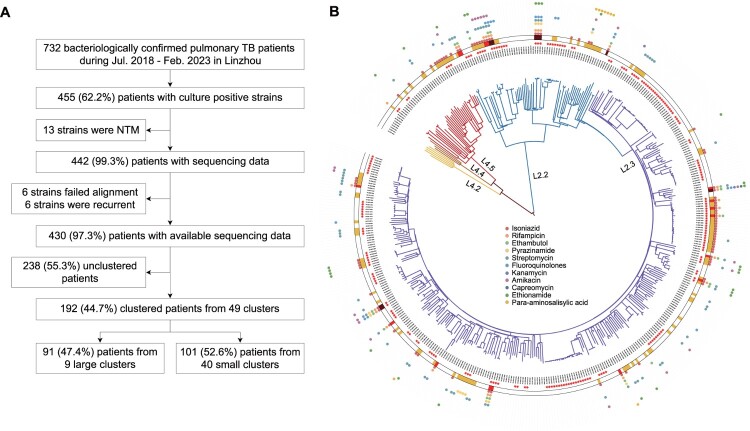
Patient enrollment and study flowchart (A). Phylogeny, clustering, and resistance profiles of 430 Mycobacterium tuberculosis strains isolated in Linzhou, 2018-2023. Blue, purple, yellow, brown and red branches indicate Lineage2.2, Lineage2.3, Lineage4.2, Lineage4.4, and Lineage4.5, respectively. Solid stars outside the phylogeny indicate genomic-clustered strains differing by ≤12 single-nucleotide polymorphisms. The outer yellow-red-brown circle indicates other drug resistance, multidrug resistance and pre-extensive drug resistance, respectively. The outermost coloured dots indicate the resistance to 11 anti-TB drugs (B).

Between July 1, 2018 and February 28, 2023, there were 732 bacteriologically confirmed pulmonary TB patients in Linzhou, of whom 455 (62.2%) were culture-positive. After excluding 13 patients whose cultures grew non-tuberculous mycobacteria, the isolates from 442 patients were genome sequenced. We further excluded 6 patients whose isolates failed sequencing and 6 patients with recurrent TB. The final analysis thus included 430 patients (Figure 1A) whose median age was 46 years (IQR, 27–64 years) and of whom 68.4% (294/430) were males. The proportion of smear-positive patients was 63.0% (271/430), 425 (98.8%) were registered as new patients and 50 (11.6%) were students (Table 1).

**Table 1.** Univariate and multivariable logistic regression of risk factors for genomic clustering.

	Total (n = 430)	Clustered (n = 192)	Unclustered (n = 238)	Univariable Regression		Multivariable Regression	
				OR (95% CI)	*P* value	aOR (95% CI)	*P* value
**Demographic factors**							
Gender							
Male	294 (68.4)	123 (41.8)	171 (58.2)	1.00		1.00	
Female	136 (31.6)	69 (50.7)	67 (49.3)	1.43 (0.95, 2.15)	0.085	1.43 (0.91, 2.24)	0.117
Age, years							
< 25	84 (19.5)	55 (65.5)	29 (34.9)	5.69 (3.02, 10.70)	<0.001	2.19 (0.95, 5.05)	0.067
25–44	126 (29.3)	67 (53.2)	59 (46.8)	3.41 (1.94, 6.00)	<0.001	3.08 (1.71, 5.55)	<0.001
45–64	116 (27.0)	44 (37.9)	72 (62.1)	1.83 (1.03, 3.28)	0.041	1.90 (1.04, 3.47)	0.038
≥65	104 (24.2)	26 (25.0)	78 (75.0)	1.00		1.00	
Occupation							
Student	50 (11.6)	39 (78.0)	11 (22.0)	5.26 (2.61, 10.59)	<0.001	4.51 (1.69, 12.02)	0.003
Others	380 (88.4)	153 (40.3)	227 (59.7)	1.00		1.00	
**Clinical factors**							
TB history							
New case	424 (98.6)	191 (45.0)	233 (55.0)	1.00		..	
Retreated case	6 (1.4)	1 (16.7)	5 (83.3)	0.24 (0.03, 2.11)	0.200	..	..
Total delay							
<2 weeks	193 (44.9)	89 (46.1)	104 (53.9)	1.00		..	
2–4 weeks	131 (30.5)	58 (44.3)	73 (55.7)	0.93 (0.59, 1.45)	0.744	..	..
4–8 weeks	73 (17.0)	31 (42.5)	42 (57.5)	0.86 (0.50, 1.49)	0.594	..	..
≥8 weeks	33 (7.7)	14 (42.4)	19 (57.6)	0.86 (0.41, 1.82)	0.694	..	..
Sputum smear status							
Negative	159 (37.0)	72 (45.3)	87 (54.7)	1.00		..	
Positive	271 (63.0)	120 (44.3)	151 (55.7)	0.96 (0.65, 1.42)	0.84	..	..
**Bacteriological factors**							
Genotype							
Modern Beijing	322 (74.9)	156 (48.5)	166 (51.6)	4.70 (2.03, 10.89)	<0.001	3.28 (1.34, 7.99)	0.009
Ancient Beijing	66 (15.3)	29 (43.9)	37 (56.1)	3.92 (1.52, 10.09)	0.005	2.97 (1.09, 8.11)	0.034
Non-Beijing	42 (9.8)	7 (16.7)	35 (83.3)	1.00		1.00	
DR profile							
Pan-susceptible	291 (67.7)	130 (44.7)	161 (55.3)	1.00		..	
Other DR	114 (26.5)	49 (43.0)	65 (57.0)	0.93 (0.60, 1.45)	0.758	..	..
MDR	25 (5.8)	13 (52.0)	12 (48.0)	1.34 (0.59, 3.04)	0.481	..	..
**Geographic factors**							
Living area							
Central Subdistrict	76 (17.7)	43 (56.6)	33 (43.4)	2.24 (1.33, 3.75)	0.002	1.77 (1.02, 3.10)	0.044
Hengshui	28 (6.5)	15 (53.6)	13 (46.4)	1.98 (0.91, 4.33)	0.087	1.81 (0.79, 4.15)	0.163
Rencun	39 (9.1)	24 (61.5)	15 (38.5)	2.75 (1.38, 5.48)	0.004	2.96 (1.41, 6.18)	0.004
Lingyang	18 (4.2)	11 (61.1)	7 (38.9)	2.70 (1.01, 7.19)	0.047	2.65 (0.95, 7.43)	0.063
Others	269 (62.5)	99 (36.8)	170 (63.2)	1.00		1.00	

### Phylogenetic structure and drug resistance of strains

The majority (90.2%, 388/430) of the strains belonged to the Beijing Lineage 2, primarily the modern Beijing sublineage L2.3 (83.0%, 322/388). All of the non-Beijing strains belonged to the Euro-American Lineage 4 (9.8%, 42/430), predominantly sublineage L4.5 (81.0%, 34/42) (Figure 1B). SAM-TB was used to predict the drug resistance profile for each strain (Table S1) and detected drug resistance-associated mutations in 139 strains (32.3%, 139/430). MDR strains accounted for 5.8% (25/430) of the total, and 36.0% (9/25) of the MDR strains also contained mutations conferring resistance to fluoroquinolones and were therefore classified as pre-XDR. No mutations associated with resistance to linezolid, clofazimine, bedaquiline or delamanid were detected.

### Clustering analysis, factors associated with genomic clustering and cause of large clusters

A total of 192 (44.7%, 192/430) patients were grouped into 49 genomic clusters containing 2–22 patients. Clusters containing two patients accounted for 51.0% (25/49) of the total clusters, while clusters with ≥5 patients constituted 18.4% (9/49) (Figure 1B, Figure S2). Multivariable logistic regression (Table 1) found that students had a greater risk of clustering (aOR 4.51, 95% CI 1.69-12.02) than non-students, and patients residing in the Central Subdistrict (aOR 1.77, 95%CI 1.02-3.10) or Rencun Township (aOR 2.96, 95%CI 1.41-6.18) had a greater risk of clustering than patients residing in other areas of Linzhou city. Increased clustering was also significantly associated with patient age (25-44 years: aOR 3.08, 95%CI 1.71-5.55; 45–64 years: aOR 1.90, 95%CI 1.04-3.47) and patients whose isolates belonged to the Beijing Lineage 2 (modern Beijing Lineage: aOR 3.28, 95%CI 1.34-7.99; ancient Beijing Lineage: aOR 2.97, 95%CI 1.09-8.11).

We then investigated whether specific patient characteristics were associated with belonging to large clusters, as has been suggested in previous studies [22,23]. Clusters containing five or more patients were defined as large and all other clusters as small [24]. Univariate logistic regression comparing the characteristics of the 91 patients belonging to the 9 large clusters, with the 101 patients belonged to the 40 small clusters found no significant differences between the two groups (Table S2). The same result was obtained when other thresholds were used to define large clusters (i.e. ≥ 4, ≥ 8, or ≥9), confirming that there were no significant risk factors associated with belonging to large clusters.

Previous studies have reported that super-spreaders, who infect multiple individuals, can generate large genomic clusters [25]. To explore whether transmission from super-spreaders could be responsible for the large genomic clusters, we performed transmission inference for each cluster. After excluding the initial transmission event to the putative index case for each cluster, we estimated that there were 144 transmission events, of which 113 (78.5%) had a posterior probability >0.5. These 113 plausible transmission events were caused by 85 index cases, of whom 92.9% (79/85) transmitted to 1–2 secondary cases and only a few transmitted their infection to cause 3–4 secondary cases (Table S3, 4). No super-spreaders were detected. This indicates that patient transmission typically engenders only a few secondary cases, but the newly infected individuals can, in turn, transmit the infection to cause additional secondary cases, thus extending the transmission chain to form large clusters (Figure S3).

### Epidemiological links of genomic-clustered patients

To delineate the transmission links between the genomic-clustered patients, we attempted to perform in-depth epidemiological investigation of all clustered patients. Eleven clustered patients refused to be investigated and 49 clustered patients had died or were lost to follow-up, allowing complete epidemiological investigations for only 68.8% (132/192) of the clustered patients. Confirmed epidemiological links were identified in 24 (18.2%, 24/132) of the clustered patients investigated, and probable epidemiological links were identified in an additional 26 (19.7%, 26/132) patients. The characteristics of each cluster with confirmed or probable epidemiological links are shown in Table S5.

All 24 patients with confirmed epidemiological links were known contacts: close family contacts were identified in 33.3% (8/24); social contacts in 66.7% (16/24), half of whom were classmates (50.0%, 8/16); villagers who knew each other (37.5%, 6/16); and relatives (12.5%, 2/16). Probable epidemiological links were most frequently found between patients who were from the same village but did not know each other (46.2%, 12/26) and students who attended the same school but did not know each other (26.9%, 7/26).

### Geographical distance and spatial hotspots analysis

**Figure 2.**
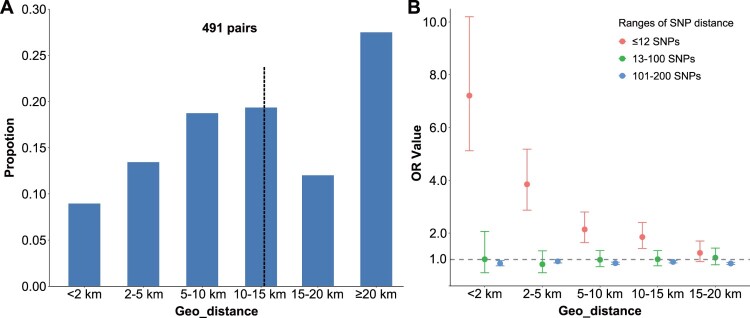
Histograms of the geographical distance between the residences of patient pairs whose MTB isolates differ by ≤12 SNPs (A). Relation of SNP difference and pairwise geographical residence distance between patient pairs, using patient pairs residing 20 or more kilometers apart as the reference (B). Different colours indicate ranges of SNP differences between pairs of MTB isolates. Error bars indicate 95% confidence intervals.

Many previous studies in urban areas have shown that geographically proximate TB patients are more likely to fall within a genomic cluster [20,26]. To determine whether there was a relationship between genomic clustering and the proximity of patient residences in rural areas, we calculated the pairwise SNP differences and the geographical distances between all possible pairwise combinations of the 417 patients who provided detailed home addresses. We found that the median geographical distance between the residences of all possible patient pairs was 18.2 km. We then analysed the geographical distance between the residences of the 491 clustered patient pairs who had strains that differed by ≤12 SNPs and found that the median geographical distance was 13.0 km. Only 9.0% (44/491) of these clustered pairs lived <2.0 km apart, and 22.4% (110/491) lived <5.0 km apart (Figure 2A). Surprisingly, 78.6% (386/491) of clustered patient pairs lived in different townships, and 36.3% (178/491) involved patients residing in the Central Subdistrict. Using univariate logistic regression to evaluate the risk for genomic clustering at different levels of geographic proximity, we found that patients living within 2 km of each other had the highest risk of clustering (OR 7.21, 95%CI 5.12-10.17), but the risk of clustering decreased rapidly as the geographic distance between patient residences increased. There was no association of residence proximity and SNP difference for patients whose isolates differed by >12 SNPs (Figure 2B, Table S6).

**Figure 3.**
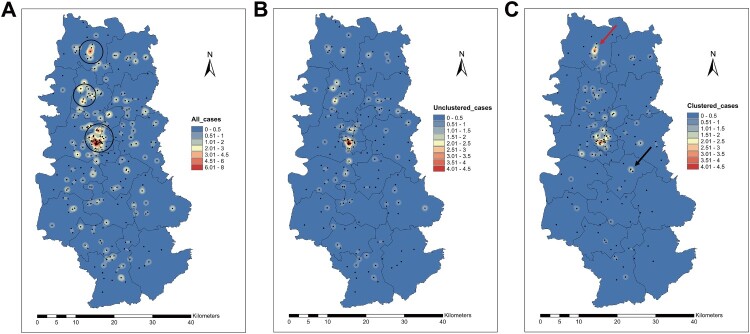
Kernel density maps of: (A), all patients; (B), unclustered patients; and (C), clustered patients. The three maps were coloured according to kernel density estimation, with red representing areas with the most intense aggregation of patients. The red and black arrows in panel C indicate the Rencun and the Caisang Township, respectively.

To identify spatial hotspots of local transmission we used the patients’ residential address to draw kernel density maps. When all patients were considered, spatial hotspots appeared in Yaocun Township, Rencun Township, and the Central Subdistrict (Figure 3A). We then drew kernel density maps separately for clustered and unclustered patients (Figure 3B, C) and found that the Central Subdistrict had the highest spatial distribution density, and was thus a hotspot for both clustered and unclustered patients. In contrast, Yaocun Township was identified as a hotspot largely due to the spatial aggregation of unclustered patients, while Rencun Township was a hotspot primarily for clustered patients. In addition, there were several other hotspots with lower spatial distribution densities in Lingyang, Hengshui, Caisang, and Huanghua Townships (Figure 3C). Further analysis revealed that in the Central Subdistrict and Rencun Township hotspots, clustered patients transmitted the disease predominantly to other individuals residing within the same township, who accounted for 69.8% (30/43) and 66.7% (16/24), respectively, of the clustered patients in these two sites. Thus, these hotspots for clustered patients appear to be high-risk areas for local TB transmission. In contrast, Caisang Township, which also had a relatively high patient density, did not appear to be a hotspot for local transmission because the eight clustered patients residing there were involved in eight different clusters that each included patients from other townships (Table S7).

### Transmission of DR-TB

DR-TB can be divided into transmitted resistance, caused by direct infection with a drug resistant strain, or acquired resistance, which develops during treatment in a previously drug sensitive strain [5]. To investigate the etiology of DR-TB in Linzhou, we defined transmitted resistance as new TB patients with resistant isolates and retreated, clustered patients with DR isolates carrying identical resistance mutations. Other resistant patients were defined as acquired resistance [27,28]. Among the 25 MDR patients, 24 were new patients and one was a retreated, unclustered patient. Thus, 96.0% (24/25) of MDR-TB patients were infected with transmitted resistant strains. Furthermore, we found that transmission of resistant strains was responsible for 96.2% (25/26) of patients with rifampicin resistance, 98.9% (86/87) with isoniazid resistance, and 100% (17/17) of patients with fluoroquinolone resistance, indicating that local transmission is the overwhelming cause of the DR-TB burden in Linzhou.

## Discussion

This study combined genomic, epidemiological, and spatial analysis to determine that 44.7% of the TB cases in Linzhou were caused by recent MTB transmission. However, even with in-depth investigation, epidemiologic connections were found in less than 40% of the clustered cases. Spatial epidemiology suggested increased clustering among patients residing within 2.0 km of each other, but most transmission occurred between patients who did not live in close proximity. Although the Central Subdistrict and Rencun Township were identified as areas at high risk of TB transmission, the median geographical distance between the residences of all clustered patient pairs was 13.0 km, and 78.6% of these pairs resided in different townships.

The overall proportion of clustered TB patients in Linzhou, 44.7%, is significantly higher than has been reported in other rural areas in China, such as Wusheng County in Sichuan Province (26.9%) [29], Wuchang City in Heilongjiang Province (39.6%) [29], Yidu and Zigui County in Hubei Province (30.1%) [30], and six counties across Shandong and Jiangsu Provinces (15.3%) [10]. Moreover, the proportion of large clusters (≥5 patients) in Linzhou is also higher (18.4%) than reported for these other sites. Together, these findings suggest that, for unknown reasons, recent, local transmission of TB is more prevalent in Linzhou than in the other sites that have been studied. In addition, the majority of clustered patient pairs resided ≥5.0 km apart, indicating that local transmission likely occurs primarily in venues of social aggregation that are not associated with patient residences.

In-depth epidemiological investigations in previous studies have suggested that an increased risk and expanded spatial scale for transmission associated with venues where people congregate for education, healthcare, entertainment, shopping or incarceration [19,29,31,32]. For example, a study conducted in Wusheng County, Sichuan Province found that 62.7% of transmission occurred among social contacts, with teahouses appearing as important sites for transmission between individuals from different villages [29]. Another study, conducted in Chiang Rai Province, Thailand, found that patients with a history of incarceration were more likely to be involved in large clusters, and the spatial scale of patients within these large clusters often spanned entire provinces [33]. Our study only identified schools as high-risk settings for transmission, with students showing the highest risk of clustering and transmission occurring between students whose residences were far apart. We also found that many of the clustered patient pairs involved patients residing in the Central Subdistrict, where many commercial, entertainment, educational, and medical facilities provide numerous opportunities for transmission between patients from different areas. Our epidemiological investigations found that residents from different townships often seek medical care in the Central Subdistrict. Furthermore, within one cluster of 15 patients (C2), 3 young patients reported frequenting entertainment settings such as karaoke bars in the Central Subdistrict. This cluster consisted almost entirely of younger patients, with only one patient over the age of 30.

Kernel density estimation identified Rencun Township as a transmission hotspot, where 66.7% of clustered patients were clustered with other patients from the same township. Rencun Township has several tourist attractions where local residents frequently gather to sell handicrafts and socialize, perhaps providing the setting for TB transmission among the local population. In contrast, Yaocun Township was identified as a hotspot largely due to the spatial aggregation of unclustered patients. Non-clustering can reflect reactivation of latent MTB infection or transmission from patients who are not local residents [34]. Thus, the interpretation of unclustered patients is complex, and factors such as study design, geographical coverage, observation period, and time interval between cases often should be taken into consideration [35]. Our study noted that the well-known Esophageal Cancer Hospital, located in Yaocun Township, is frequently visited by residents from other cities, who may have been infected with TB and transmitted their infections to the local residents.

However, spatial hotspots with a relatively high distribution density for clustered patients do not necessarily indicate increased transmission amongst the residents of these areas [20,36]. For example, Caisang Township appeared as a spatial hotspot for clustered patients, but the eight clustered patients in this area were not clustered with each other but rather belonged to eight different clusters that included patients from other areas. It could, therefore, be a hotspot for transmission between the local residents and people coming to Caisang from other townships.

The current limitations in interpreting transmission links are not specific to this study, but reflect significant challenges in genomic epidemiology [37,38]. Consistent with our findings, many previous studies have shown that close contacts account for only a minority of transmission, especially in high-burden settings [26,31,39]. In Malawi, population-based WGS revealed that only 9.4% of transmission occurred between close contacts [39]. Similarly, studies from South Africa [31] and China [26] found that a high proportion of clustered patients identified by WGS lacked confirmed epidemiological links. The scarcity of confirmed epidemiological links can be attributed to variations in the onset times of cases, spatial migrations, transmission from casual contact with asymptomatic patients [40] and the inherent challenge of identifying all potential contacts [41]. Patients can develop the disease months or years after they were infected, hindering the identification of epidemiological links and interpretation of transmission dynamics. Additionally, incomplete case capture can also limit the ability to accurately identify epidemiological links [38].

This study had several limitations, the most important limitation was a likely underestimate of clustering and the extent of local TB transmission. Some patients could be misclassified as unclustered when they were, in fact, clustered with patients in the same transmission chain who were diagnosed outside the study’s temporal or geographical limits. Another limitation was that geographical data was based only on the patients’ residence at the time of diagnosis and did not include information on their previous residences or other venues they frequented. This could have affected the calculation of geographical distances between patient pairs and the identification of spatial hotspots.

In conclusion, recent, local transmission is an important driver of the TB burden in Linzhou. Much of the transmission appears to be associated with settings where the rural populations congregate – schools, tourist attractions, entertainment venues, etc. Further studies are needed to identify additional high-risk populations and the local venue-based amplifiers for transmission in rural areas. These insights will offer theoretical guidance for improved prevention and control strategies to reduce the incidence of TB in China’s rural communities.

## Author contributions

Q.J., ZH.R. and Q.G. designed and managed the study. Z.Q., JY.X., M.L., CY.C., P.M. and ZH.R. performed the epidemiological investigation; Z.Q. and M.L. cleaned the data and performed statistical analysis; Z.Q. and M.L. performed the sequence analysis and interpretation. Z.Q., M.L., Q.J., H.T. and Q.G. prepared the manuscript. All authors contributed to and gave input to the final version of the manuscript.

## Acknowledgements

We thank the tuberculosis public health teams in the Linzhou City Centre for Disease Control and Prevention.

## Supplementary Material

Supplementary_Table_S3.xlsx

Supplementary_material.docx

## Data Availability

Sequencing data were deposited in the Genome Sequence Archive (https://bigd.big.ac.cn/gsa) under BioProject PRJCA025885. De-identified participant data from the study will be made available upon publication to medical researchers on a not-for-profit basis by email request to the corresponding author for the purposes of propensity matching or meta-analysis.
