# Supplementary material for: Transmission of tuberculosis in rural Henan, China: a prospective population-based genomic spatial epidemiological study
Source: Emerg Microbes Infect. 2024 Aug 29;13(1):2399273. doi: 10.1080/22221751.2024.2399273 (PMC11378662; doi:10.1080/22221751.2024.2399273)
Supplement: Supplementary_material.docx [file TEMI_A_2399273_SM2118.docx]

**Supplementary material**

**Transmission of Tuberculosis in Rural Henan, China: A Prospective Population-based Genomic Spatial Epidemiological Study**

**Content**

| **Table S1.** Drug resistance in 430 Pulmonary TB patients diagnosed in Linzhou, Henan, 2018-2023 and included in the study. | Page 2 |
| --- | --- |
| **Table S2.** Analysis of patient characteristics in large vs. small clusters in Linzhou. | Page 3 |
| **Table S4.** Number of secondary cases resulting from 192 clustered cases based on transmission inference analysis. | Page 4 |
| **Table S5.** Characteristics of genomic clusters with confirmed (24) or probable (26) epidemiological links. | Page 5 |
| **Table S6.** Distribution of patient pairs by the spatial distance between the patient residences and the SNP distances between their isolates, using patient pairs residing 20 or more kilometers apart as reference. | Page 6 |
| **Table S7.** Patient clusters in spatial hotspots. | Page 7 |
| **Figure S1.** Administrative divisions (A) and reported incidence of tuberculosis of Linzhou (B). | Page 8 |
| **Figure S2.** Number of genomic clusters by cluster size (A). The proportion of large and small clusters in six different regions of China (B). | Page 9 |
| **Figure S3.** Diagnosis timeline of the 91 total patients in the 9 large clusters with ≥5 patients each. | Page 10 |

**Table S1.** Drug resistance in 430 Pulmonary TB patients diagnosed in Linzhou, Henan, 2018-2023 and included in the study.

| Drug resistance | No. of Isolates (%) |
| --- | --- |
| Isoniazid | 87 (20.2) |
| Rifampicin | 26 (6.0) |
| Ethambutol | 19 (4.4) |
| Pyrazinamide | 13 (3.0) |
| Streptomycin | 71 (16.5) |
| Fluoroquinolones (Ofloxacin/Levofloxacin; Moxifloxacin) | 17 (4.0) |
| Kanamycin | 2 (0.5) |
| Amikacin | 21 (4.9) |
| Capreomycin | 1 (0.2) |
| Ethionamide | 27 (6.3) |
| Para-aminosalicylic acid | 9 (2.1) |
| Cycloserine | 0 |
| Linezolid | 0 |
| Clofazimine | 0 |
| Bedaquiline | 0 |
| Delamanid | 0 |
| Multidrug resistance | 25 (5.8) |
| Pre-extensive drug-resistant | 9 (2.1) |

**Table S2.** Analysis of patient characteristics in large vs. small clusters in Linzhou. Clusters containing ≥5 patients are regarded as large.

|  | Large cluster of patients (n=91) | Small cluster of patients (n=101) | Univariable Regression | |
| --- | --- | --- | --- | --- |
|  |  |  | OR (95% CI) | *P* value |
| **Demographic factors** | | | | |
| Gender | | |  |  |
| Male | 62 (50.4) | 61 (49.6) | 1.00 |  |
| Female | 29 (42.0) | 40 (58.0) | 0.71 (0.39, 1.29) | 0.265 |
| Age, years | | |  |  |
| < 25 | 24 (43.6) | 31 (56.4) | 0.90 (0.35, 2.31) | 0.831 |
| 25-45 | 38 (56.7) | 29 (43.3) | 1.53 (0.62, 3.80) | 0.361 |
| 45-64 | 17 (38.6) | 27 (61.4) | 0.73 (0.28, 1.96) | 0.538 |
| ≥65 | 12 (46.2) | 14 (53.9) | 1.00 |  |
| Occupation | | |  |  |
| Student | 15 (38.5) | 24 (61.5) | 0.63 (0.31, 1.30) | 0.213 |
| Others | 76 (49.7) | 77 (50.3) | 1.00 |  |
| **Clinical factors** | | | | |
| TB history | | |  |  |
| New case | 91 (47.6) | 100 (52.4) | 1.00 |  |
| Retreated case | 0 | 1 (100.0) | .. | .. |
| Total delay | | |  |  |
| <2 weeks | 41 (46.1) | 48 (53.9) | 1.00 |  |
| 2-4 weeks | 30 (51.7) | 28 (48.3) | 1.25 (0.65, 2.43) | 0.503 |
| 4-8 weeks | 13 (41.9) | 18 (58.1) | 0.85 (0.37, 1.93) | 0.691 |
| ≥8 weeks | 7 (50.0) | 7 (50.0) | 1.17 (0.38, 3.62) | 0.784 |
| Sputum smear status | | | |  |
| Negative | 36 (50.0) | 36 (50.0) | 1.00 |  |
| Positive | 55 (45.8) | 65 (54.2) | 0.85 (0.47, 1.52) | 0.576 |
| **Geographic factors** | | | | |
| Living area | | |  |  |
| Central subdistrict | 20 (46.5) | 23 (53.5) | 0.89 (0.43, 1.82) | 0.744 |
| Hengshui | 5 (33.3) | 10 (66.7) | 0.51 (0.16, 1.60) | 0.249 |
| Rencun | 11 (45.8) | 13 (54.2) | 0.86 (0.35, 2.11) | 0.748 |
| Lingyang | 6 (54.6) | 5 (45.4) | 1.22 (0.35, 4.28) | 0.751 |
| Others | 49 (49.5) | 50 (50.5) | 1.00 |  |

**Table S4.** Number of secondary cases resulting from 192 clustered cases based on transmission inference analysis. Transmission events with posterior probability >0.5 were included in the analysis.

| No. of secondary cases | No. of clustered cases (*P* >0.5) |
| --- | --- |
| ≥5 | 0 |
| 4 | 1 (1.2%) |
| 3 | 5 (5.9%) |
| 2 | 15 (17.6%) |
| 1 | 64 (75.3%) |
| 0 | 107 |

**Table S5.** Characteristics of genomic clusters with confirmed (24) or probable (26) epidemiological links.

|  | Number of cases in clusters | Mean age (years [IQR]) | Number of patients living in the same village | Number of new cases | Number of patients receiving investigation | Epidemiologically linked | Nature of epidemiological link [number of patients] |
| --- | --- | --- | --- | --- | --- | --- | --- |
| Cluster01 | 22 | 39 | 4 | 22 | 15 | Probable | same village [4] |
| Cluster02 | 15 | 25 | 2 | 15 | 11 | Confirmed/Probable | classmate [2]; friend [2]; same village [2] |
| Cluster03 | 10 | 34 | 2 | 10 | 7 | Probable | same village [2] |
| Cluster04 | 9 | 56 | 2 | 9 | 5 | Confirmed | same village and knew each other [2] |
| Cluster05 | 9 | 33 | 2 | 9 | 6 | Confirmed | same village and knew each other [2] |
| Cluster06 | 8 | 34 | 2 | 8 | 7 | Probable | same community [2] |
| Cluster08 | 5 | 39 | 2 | 5 | 4 | Confirmed/Probable | same village and knew each other [2]; workplace [3] |
| Cluster09 | 5 | 30 | 0 | 5 | 2 | Probable | same school [2] |
| Cluster13 | 4 | 17 | 0 | 4 | 3 | Confirmed/Probable | classmate [2]; same school [2] |
| Cluster15 | 4 | 16 | 0 | 4 | 3 | Probable | same school [2] |
| Cluster17 | 3 | 20 | 0 | 3 | 2 | Probable | same school [2] |
| Cluster20 | 3 | 33 | 0 | 3 | 2 | Confirmed | classmate [2] |
| Cluster21 | 3 | 32 | 0 | 3 | 2 | Confirmed | classmate [2] |
| Cluster22 | 3 | 49 | 0 | 3 | 2 | Probable | knew same people and probably contact [2] |
| Cluster23 | 3 | 48 | 2 | 3 | 2 | Confirmed | family [2] |
| Cluster32 | 2 | 37 | 2 | 2 | 2 | Probable | same village [2] |
| Cluster36 | 2 | 38 | 0 | 2 | 1 | Confirmed | family [2] |
| Cluster42 | 2 | 20 | 2 | 2 | 2 | Confirmed | family [2] |
| Cluster43 | 2 | 38 | 2 | 2 | 2 | Confirmed | relative [2] |
| Cluster47 | 2 | 36 | 2 | 2 | 1 | Confirmed | family [2] |
| Cluster49 | 2 | 47 | 0 | 2 | 2 | Probable | knew same people and probably contact [2] |

**Table S6.** Distribution of patient pairs by the spatial distance between the patient residences and the SNP distances between their isolates, using patient pairs residing 20 or more kilometers apart as reference.

|  | >12 SNPs | ≤12 SNPs | Odds ratio | Others | 13-100 SNPs | Odds ratio | Others | 101-200 SNPs | Odds ratio | Total |
| --- | --- | --- | --- | --- | --- | --- | --- | --- | --- | --- |
| Total | 86245 | 491 |  | 86355 | 381 |  | 68235 | 18501 |  | 86736 |
| <2 km | 1755 (97.55) | 44 (2.45) | 7.21 (5.12, 10.17) | 1791 (99.56) | 8 (0.44) | 1.01 (0.50, 2.06) | 1441 (80.10) | 358 (19.98) | 0.85 (0.76, 0.96) | 1799 |
| 2-5 km | 4928 (98.68) | 66 (1.32) | 3.85 (2.87, 5.18) | 4976 (99.64) | 18 (0.36) | 0.82 (0.50, 1.33) | 3928 (78.65) | 1066 (21.35) | 0.93 (0.87, 1.00) | 4994 |
| 5-10 km | 12348 (99.26) | 92 (0.74) | 2.14 (1.64, 2.80) | 12386 (99.57) | 54 (0.43) | 0.99 (0.73, 1.34) | 9961 (80.07) | 2479 (19.93) | 0.86 (0.81, 0.90) | 12440 |
| 10-15 km | 14791(99.36) | 95 (0.64) | 1.85 (1.42, 2.40) | 14820 (99.56) | 66 (0.44) | 1.01 (0.76, 1.34) | 11763 (79.02) | 3123 (20.98) | 0.91 (0.87, 0.96) | 14886 |
| 15-20 km | 13578 (99.57) | 59 (0.43) | 1.25 (0.92, 1.70) | 13573 (99.53) | 64 (0.47) | 1.07 (0.80, 1.43) | 10939 (80.22) | 2698 (19.78) | 0.85 (0.81, 0.89) | 13637 |
| ≥20 km | 38845 (99.65) | 135 (0.35) | 1.00 | 38809 (99.56) | 171 (0.44) | 1.00 | 30203 (77.48) | 8777 (22.52) | 1.00 | 38980 |

**Table S7.** Patient clusters in spatial hotspots.

| Living area | No. of patients | No. of clustered patients (%) | No. of patients clustered with others in same township (%) |
| --- | --- | --- | --- |
| Centre Subdistrict | 76 | 43 (56.6) | 30 (69.8) |
| Rencun | 39 | 24 (61.5) | 16 (66.7) |
| Lingyang | 18 | 11 (61.1) | 6 (54.5) |
| Huanghua | 29 | 13 (44.8) | 7 (53.8) |
| Hengshui | 28 | 15 (53.6) | 6 (40.0) |
| Caisang | 18 | 8 (44.4) | 0 |


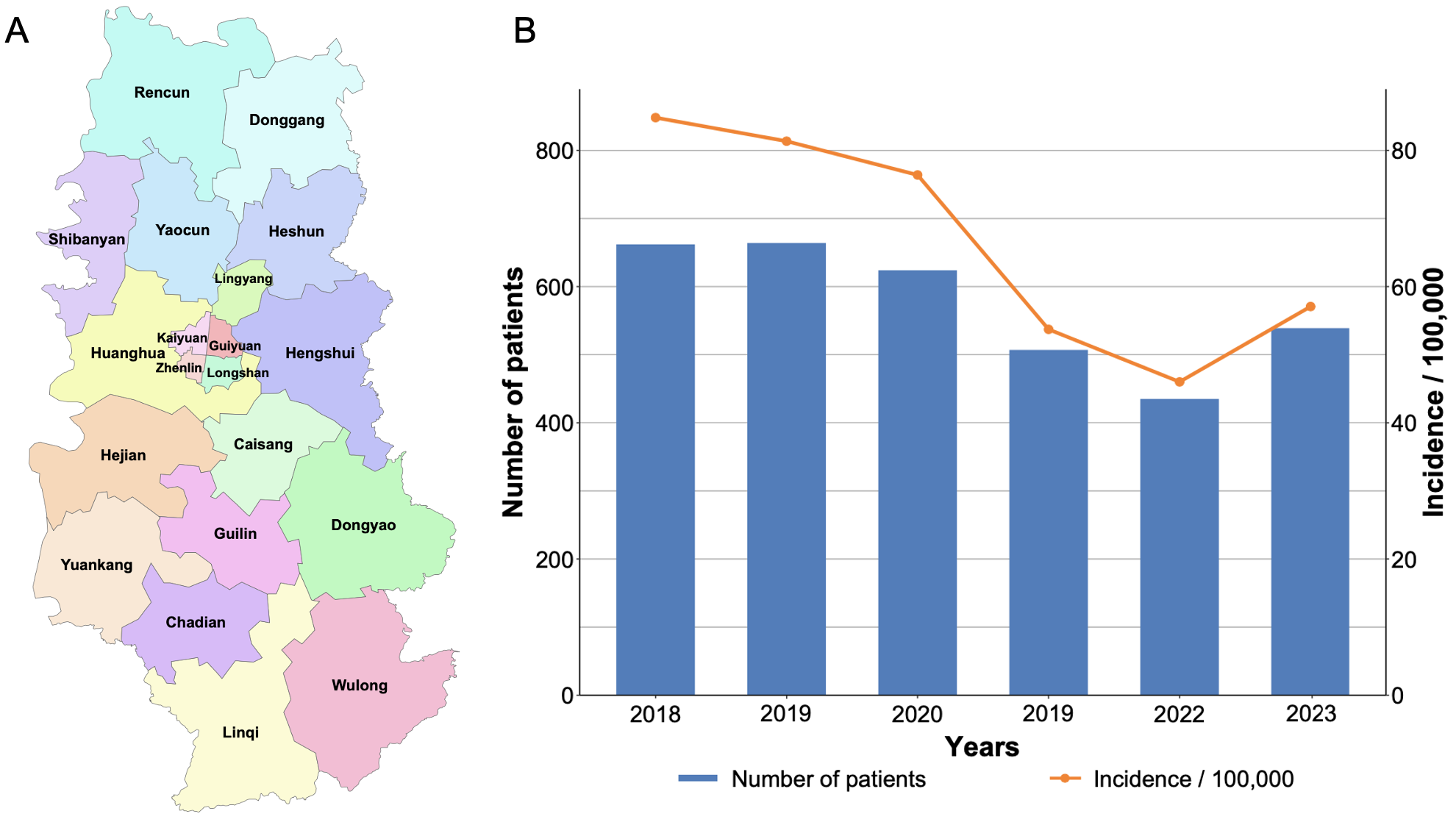


**Figure S1.** Administrative divisions (A) and reported incidence of tuberculosis of Linzhou (B). Kaiyuan, Guiyuan, Zhenlin, and Longshan Subdistrict are regarded as the Central Subdistrict.


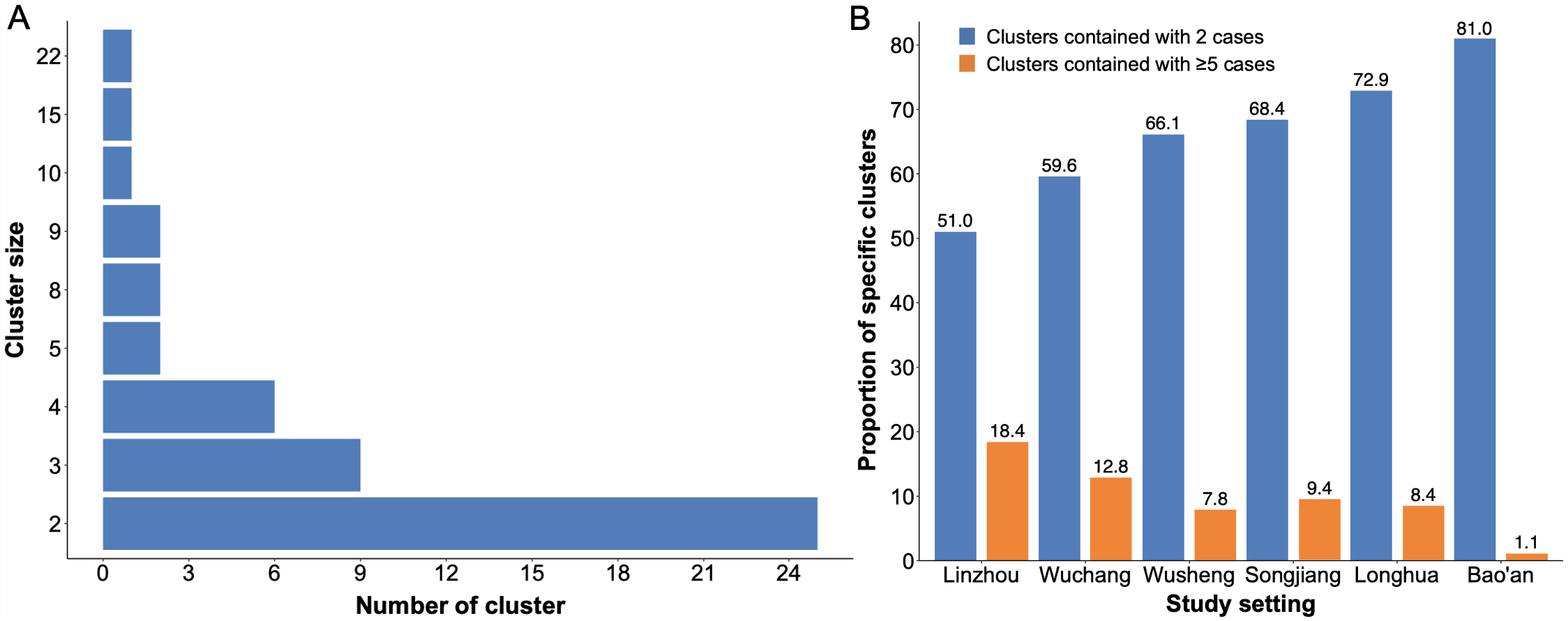


**Figure S2.** Number of genomic clusters by cluster size (A). The proportion of large and small clusters in six different regions of China (B). The results of other five regions have been reported [1-4].


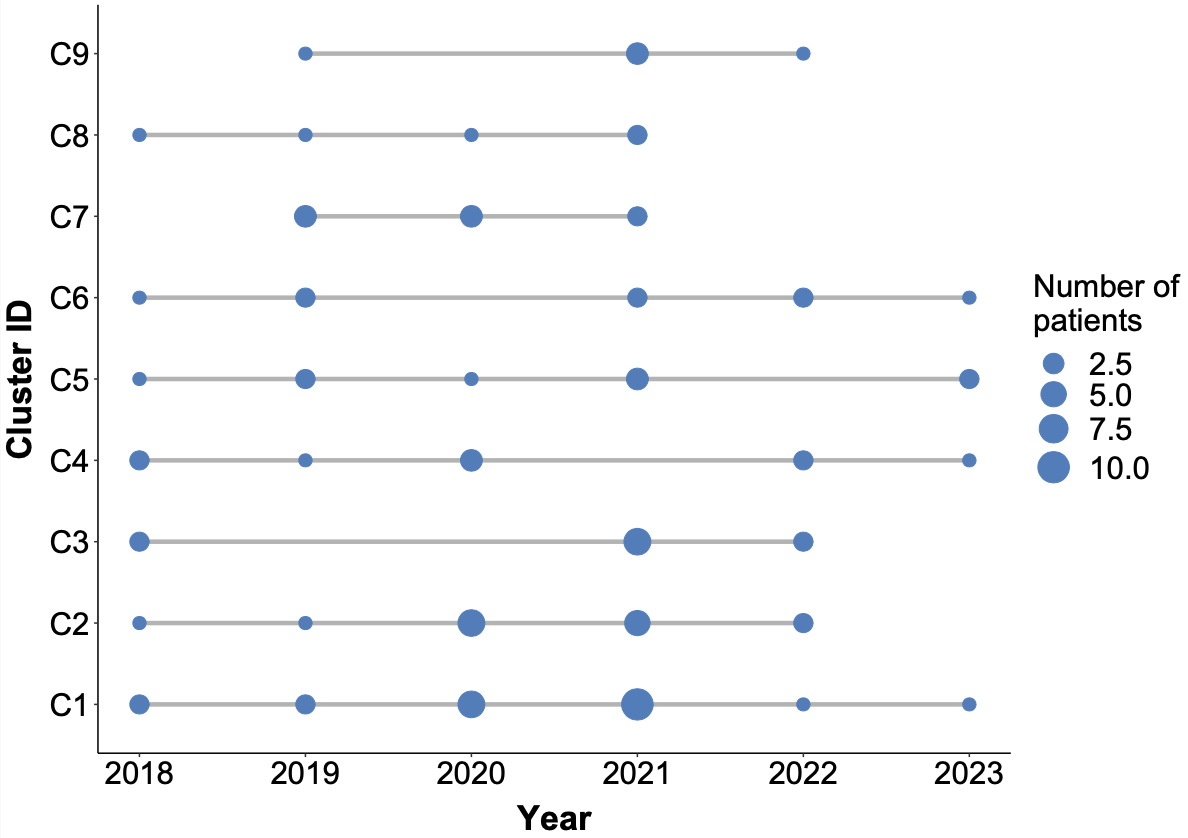


**Figure S3.** Diagnosis timeline of the 91 total patients in the 9 large clusters with ≥5 patients each.

**Reference**

[1] Yang T, Wang Y, Liu Q, et al. A population-based genomic epidemiological study of the source of tuberculosis infections in an emerging city: Shenzhen, China. Lancet Reg Health West Pac. 2021 Mar;8:100106.

[2] Li M, Guo M, Peng Y, et al. High proportion of tuberculosis transmission among social contacts in rural China: a 12-year prospective population-based genomic epidemiological study. Emerg Microbes Infect. 2022 Dec;11(1):2102-2111.

[3] Li M, Lu L, Jiang Q, et al. Genotypic and spatial analysis of transmission dynamics of tuberculosis in Shanghai, China: a 10-year prospective population-based surveillance study. Lancet Reg Health West Pac. 2023 Sep;38:100833.

[4] Mijiti P, Liu C, Hong C, et al. Implications for TB control among migrants in large cities in China: A prospective population-based genomic epidemiology study in Shenzhen. Emerg Microbes Infect. 2023 Nov 22;13(1):2287119.
